# The Conditioning Lesion Response in Dorsal Root Ganglion Neurons Is Inhibited in Oncomodulin Knock-Out Mice

**DOI:** 10.1523/ENEURO.0477-21.2022

**Published:** 2022-02-23

**Authors:** Jon P. Niemi, Talia DeFrancesco-Oranburg, Andrew Cox, Jane A. Lindborg, Franklin D. Echevarria, Jemima McCluskey, Dwayne D. Simmons, Richard E. Zigmond

**Affiliations:** 1 Case Western Reserve University, Cleveland, OH 44106-4975; 2 Department of Biology, Baylor University, Waco, TX 76798

**Keywords:** axotomy, conditioning lesion, dorsal root ganglion, macrophage, oncomodulin, regeneration

## Abstract

Regeneration can occur in peripheral neurons after injury, but the mechanisms involved are not fully delineated. Macrophages in dorsal root ganglia (DRGs) are involved in the enhanced regeneration that occurs after a conditioning lesion (CL), but how macrophages stimulate this response is not known. Oncomodulin (Ocm) has been proposed as a proregenerative molecule secreted by macrophages and neutrophils, is expressed in the DRG after axotomy, and stimulates neurite outgrowth by DRG neurons in culture. Wild-type (WT) and *Ocm* knock-out (KO) mice were used to investigate whether Ocm plays a role in the CL response in DRG neurons after sciatic nerve transection. Neurite outgrowth was measured after 24 and 48 h in explant culture 7 d after a CL. Sciatic nerve regeneration was also measured *in vivo* 7 d after a CL and 2 d after a subsequent sciatic nerve crush. The magnitude of the increased neurite outgrowth following a CL was significantly smaller in explants from *Ocm* KO mice than in explants from WT mice. *In vivo* after a CL, increased regeneration was found in WT animals but not in KO animals. Macrophage accumulation and levels of *interleukin-6* (*IL-6*) mRNA were measured in axotomized DRG from WT and *Ocm* KO animals, and both were significantly higher than in sham-operated ganglia. At 6 h after axotomy, *Il*-6 mRNA was higher in WT than in *Ocm* KO mice. Our data support the hypothesis that Ocm plays a necessary role in producing a normal CL response and that its effects possibly result in part from stimulation of the expression of proregenerative macrophage cytokines such as IL-6.

## Significance Statement

Peripheral neurons are capable of regeneration after axotomy. Regeneration is enhanced if a conditioning lesion (CL) precedes a test lesion. This CL effect seen in explant cultures does not occur if macrophage accumulation after injury is blocked; however, the mechanism underlying this macrophage effect is not known. To determine whether the macrophage cytokine oncomodulin (Ocm) is involved in this effect, wild-type (WT) and *Ocm* knock-out (KO) mice were examined. The CL effect was inhibited in explants from *Ocm* KO animals. Experiments performed *in vivo* in these two genotypes showed a clear CL effect in the WT mice but none in the *Ocm* KO animals.

## Introduction

Neurons in the peripheral nervous system can regenerate after injury; however, much remains to be clarified about the mechanisms involved. Regeneration is not a neuron autonomous process but instead is influenced by interactions between neurons and both glial cells and immune cells. After axotomy, macrophages play a well-known phagocytic role in the distal nerve segment, clearing discarded myelin and axonal debris (Gaudet et al., 2011; Rotshenker, 2011). In addition, macrophages accumulate around axotomized neuronal cell bodies, which has been shown to play a crucial role in nerve regeneration (Kwon et al., 2013; Niemi et al., 2013) and possibly in neuropathic pain (Yu et al., 2020).

A conditioning lesion (CL) has been shown to enhance outgrowth both in explant culture (Edström et al., 1996) and *in vivo* (McQuarrie and Grafstein, 1973). Subsequent studies have sought to define the neuron-macrophage interaction and identify critical molecules that are involved in the CL response. The chemokine CCL2, and perhaps other macrophage chemokines, bring CCR2+ monocytes from the bloodstream into the axotomized dorsal root ganglia (DRGs), where they differentiate into macrophages, and promote the CL response (Niemi et al., 2013). What the exact interaction is between the accumulating macrophages and neurons that results in the promotion of regeneration is unclear, although there are many candidate molecules expressed by macrophages that could be involved (Benowitz and Popovich, 2011).

One candidate is oncomodulin (Ocm), an EF-hand Ca^2+^ buffer protein, which was originally detected in rat hepatoma cells (MacManus et al., 1983; Climer et al., 2019). Ocm has since been found in outer hair cells of the ear (Sakaguchi et al., 1998; Yang et al., 2004), in macrophages (Yin et al., 2006, 2009), and in neutrophils (Kurimoto et al., 2013). The idea that Ocm might promote regeneration arose from studies on the regeneration of axons in the optic nerve in response to an inflammatory reaction in the eye. In this context, Ocm was identified as a macrophage-derived growth factor (Yin et al., 2006). When retinal ganglion cells were exposed in culture to Ocm and forskolin, neurite outgrowth was stimulated (Yin et al., 2006). Within a day after initiating inflammation in the eye with zymosan (a yeast cell wall protein-carbohydrate complex), the infiltrating immune cells showed high levels of *Ocm* mRNA and protein, the latter of which was secreted and bound to retinal ganglion cells. Inflammation-induced stimulation of axon growth following optic nerve crush was significantly reduced by two Ocm-blocking reagents. In addition to macrophages, neutrophils express Ocm, and they are the first responders during inflammation and after injury (Kurimoto et al., 2013; Fine et al., 2020).

However, the importance of Ocm in the context of optic nerve regeneration has been contested. Hauk and colleagues found that Ocm did not increase after lens injury or zymosan treatment and that substantial depletion of macrophages in the eye during inflammation did not prevent regeneration (Hauk et al., 2008). Additionally, they reported that gp130 cytokines such as ciliary neurotrophic factor (CNTF), leukemia inhibitory factor (LIF), and interleukin-6 (IL-6) secreted by glial cells in the retina are more important in stimulating regeneration than Ocm (Leibinger et al., 2009, 2013; Fischer and Leibinger, 2012). Thus, questions have been raised about the importance of Ocm in nerve regeneration.

Three studies examined a role for Ocm in the context of injury to DRG neurons. Yin et al. (2006) found that addition of Ocm to DRG neurons in dissociated cultures produced an increase in neurite outgrowth. Harel et al. (2012) reported what they described as a “meager” effect of Ocm plus dibutyryl cAMP on the growth of DRG neurons across the dorsal root entry zone after a dorsal root nerve crush. Kwon et al. (2013) found an increase in *Ocm* mRNA in the DRG 7 d after a sciatic nerve transection and found that a neutralizing antibody to Ocm blocked stimulation of outgrowth by DRG neurons following coculture with macrophages.

Surprisingly there have been no studies of nerve regeneration in *Ocm* knock-out (KO) mice. In addition, there have been no experiments published on Ocm’s role in regeneration of the peripheral process of DRG neurons *in vivo*. Here, an *Ocm* KO mouse is used to analyze the importance of this protein in the CL response of DRG neurons.

## Materials and Methods

### Animals and surgeries

Eight- to 12-week-old mice with a targeted deletion of exons 2–4 of the *Ocm* coding sequence were used for this investigation. These *Ocm* KO mice are designated Actb^cre^Ocm^flox/flox^ (Tong et al., 2016). Briefly (as described in Tong et al., 2016), *Ocm* KO mice were generated by inserting a LoxP site 5′ of exon 2 and Flp–neo–Flp–LoxP cassette was inserted 3′ of exon 4 in BAC DNA. The vector was electroporated into ES cells and clones resistant to G418 were isolated and checked for homologous recombination by Southern blot analysis. Two male clones were injected into C57BL/6J blastocysts. High-percentage chimeras were crossed with CBA/CaJ mice, and the pups were checked for germ-line transmission using Southern blottings. PCR primers used for genotyping were made from the deleted region (5′-CTC CAC ACT TCA CCA AGC AG-3′ and 5′-GCT TGG GGA CCC CCT GTC TTC A-3′) and from the targeting vector (5′-CTC CAC ACT TCA CCA AGC AG-3′ and 5′-TTT CAT GTT CAG GGA TCA AGT G-3′). The neo gene was removed when generating the Ocm heterozygote to avoid any possible interference. Ocm^flox/flox^ mice were generated and crossed with β-actin Cre mice (strain 003376; The Jackson Laboratory) to generate β-actin^Cre^Ocm^flox/flox^ mice (Ocmtm1.1Ddsi, MGI:97 401), referred to as *Ocm* KO mice. For these studies, the *Ocm* KO mice were backcrossed onto the CBA/CaJ strain (99% congenicity), and wild-type (WT) and mutant *Ocm* KO littermates were used. Additional age matched WT mice were acquired from The Jackson Laboratory (CBA/CaJ) when needed. The animals were housed three to five per cage under a 12/12 h light/dark cycle with *ad libitum* access to food and water. In the mouse, neurons in lumbar DRG L3, L4, and L5 project into the sciatic nerve (Rigaud et al., 2008). Two lesion protocols were followed. In one, the sciatic nerve was transected unilaterally under isoflurane anesthesia, and a 2-mm piece of the distal nerve segment was removed to prevent regeneration. The contralateral nerve was exposed but not transected, and the corresponding ganglia served as sham-operated controls. The animals were killed by CO_2_ inhalation 7 d later, and L3 and L4 DRGs and sciatic nerves were removed for neurite outgrowth studies, flow cytometry, and molecular biology. In other experiments, the sciatic nerve was transected unilaterally anterior to its trifurcation, and 7 d later the nerve was crushed at the level of the sciatic notch. Two days after the second lesion (i.e., the test lesion), the ipsilateral DRG and the sciatic nerve distal to the crush site were analyzed. The contralateral sciatic nerve was exposed and then 7 d later received a crush lesion. Case Western Reserve University’s Institutional Animal Care and Use Committee approved all surgical procedures.

### DRG explants

To assess the outgrowth in response to injury of sensory neurons in culture while maintaining the ganglion’s *in vivo* tissue architecture, neurite outgrowth was evaluated in explanted ganglia from WT and *Ocm* KO mice after a CL. Seven days after unilateral sciatic nerve transection, axotomized and sham-operated L5 DRGs were removed, desheathed, placed on coverslips, and overlaid with 7.5-μl Matrigel (Becton Dickinson). Culture plates were placed in a 37°C incubator for 5 min to allow gelling of the Matrigel before adding 1 ml F12 medium with the following additives: 0.5% bovine serum albumin (Jackson ImmunoResearch), 1% penicillin/streptomycin (Thermo Fisher Scientific, Invitrogen), 5 μg/ml insulin (Millipore Sigma), 630 ng/ml progesterone (Millipore Sigma), 5 ng/ml selenium (Millipore Sigma), 9 μg/ml putrescine (Millipore Sigma), and 100 μg/ml transferrin (BD Biosciences). Phase-contrast images of neurite outgrowth from each DRG were captured at 24 and 48 h after explantation using an Axiovert 405 M microscope at 10× magnification. Neurite outgrowth was assessed using MetaMorph by measuring the distance between the edge of the ganglion and the leading tip of the longest 20 processes in each explant. The length of these 20 neurites were averaged for each ganglion. Five sham-operated and five axotomized ganglia were analyzed for each genotype. At 48 h, explants were fixed and labeled with an antibody against β III tubulin (1:500; Promega; RRID:AB_430874) and the outgrowth was photographed.

### *In vivo* CL studies and regeneration analysis

Seven days after a distal unilateral sciatic nerve transection, both sciatic nerves were crushed more proximal to the DRG than the initial nerve transection site. The nerves were crushed for 45 s with ultra-fine hemostats (Fine Science Tools) at the level of the hip. Two days after nerve crush, the animals were killed by CO_2_ inhalation, and sciatic nerves and L4 DRG were removed for immunohistochemical analysis. Sciatic nerves were removed, cleaned, pinned down straight in a 35 mm dish, and fixed by immersion in 4% paraformaldehyde (PFA). Nerves were cryoprotected in 30% sucrose, embedded in Tissue-Tek O.C.T. (Electron Microscopy Sciences), and sectioned. After blocking, 60-μm sections were incubated with an antibody to SCG10 (1:4000; Novus Biologicals; RRID:AB_10011569) overnight at 4°C and then incubated in Alexa Fluor 555 secondary antibodies (1:400; Thermo Fisher Scientific, RRID:AB_162543). Nerves were imaged on a Leica SP8 confocal. The images underwent despeckling in ImageJ before SCG10 quantification. The regeneration index was measured based on the method of Shin et al. (2014). Briefly, the amount of fluorescence was assessed using MetaMorph in a 100-pixel-wide rectangle spanning the width of the nerve where it had been crushed, which was identified by transferring fluorescent microspheres from the tip of the hemostat at the time of the crush (not shown). Another rectangle was placed where the amount of fluorescence was 50% of that at the crush site. The distance between these two rectangles was measured and expressed as the regeneration index. One section from a crush only animal and one from a conditioned animal, and six to eight animals for each genotype were analyzed. Crush only indicates a nerve harvested 2 d after a crush injury, and conditioned indicates a nerve harvested 9 d after a CL and 2 d after a crush injury. An additional measure of regeneration was also quantified by measuring the percent area stained by an antibody to SCG10 at 500-μm intervals distal to the crush site and normalizing to the measured percent area stained at the crush site as performed by Weng et al. (2017). These data were represented as a regenerative ratio at each distance.

### Macrophage accumulation *in vivo*

The accumulation of macrophages in the sciatic nerve and L5 DRG after an *in vivo* CL followed by a nerve crush versus a nerve crush alone was determined. Sciatic nerve sections (20 μm) and DRG sections (10 μm) from WT and *Ocm* KO mice were incubated overnight at 4°C in an antibody to CD68 (1:200; Bio-Rad; RRID:AB_322219) and then incubated in Cy3 secondary antibodies (1:400; Jackson ImmunoResearch; RRID:AB_2340619) for 1 h followed by 4′,6-diamidino-2-phenylindole (DAPI). Images were captured at 10× (sciatic) or 25× (DRG) magnification using HCImage (Hamamatsu Corporation) then quantified using MetaMorph. Macrophage cell counts were acquired by counting the number of CD68+ cells containing DAPI using the ImageJ cell counter. For macrophage quantification in the DRG, only areas of tissue containing neuronal cell bodies were analyzed. The total number of cells across three images for each sample was calculated and then averaged for each experimental group. One section from each crush only and each conditioned animal from five (sciatic) or five to six (DRG) animals for each genotype were analyzed.

### Flow cytometry

Flow cytometry was performed on pooled L3 and L4 DRG or L3, L4, and L5 DRG after explant culture or after a test lesion *in vivo*, respectively. Explant and *in vivo* CL DRGs were enzymatically digested in 0.125% collagenase for 1 h at 37°C. Mechanical dissociation using a 23-gauge needle attached to a 1-ml syringe produced single-cell suspensions, which were filtered through a 35-μm cell strainer. For all cell suspensions, dead cells were labeled using Live Dead Fixable Blue Dead Cell Stain kit (Invitrogen, catalog #L23105) for 30 min at 4°C. Cells were then washed in FACS buffer (PBS, 1% BSA) and blocked with a monoclonal antibody to CD16/CD32 (1:500; eBioscience, RRID:AB_467133) for 10 min at 4°C. Cells were incubated with fluorophore-conjugated antibodies against CD11b (1:400, Biolegend; RRID:AB_312789) and F4/80 (1:400, Biolegend, RRID:AB_2293450) and Ly6G (1:400, Biolegend, RRID:AB_1134159) for 1 h at 4°C. Cells were washed and resuspended on a shaker in fixation buffer (2% PFA in PBS) for 15 min at room temperature. For flow experiments in which neurons were examined, the cells were then washed in PBS and resuspended in permeabilization buffer (0.7% Tween 20 in PBS) on a shaker for 15 min at room temperature. Cells were then incubated with a fluorophore-conjugated antibody against β III tubulin to label neurons (1:80, Biolegend, RRID:AB_2563609) for 30 min at room temperature in the dark. Cells were subsequently washed in FACS buffer and then run on a BD FACSAria (BD Biosciences) and analyzed using FlowJo (Tree Star, RRID:SCR_008520). All events were gated based on viable single cells. Compensation and gating were performed using negative, single-stained and isotype controls. Cell populations were gated as follows: F4/80^+^CD11b^+^ (macrophages); β-tubulin^+^CD11b^–^ (neurons), and Ly6G^+^CD11b^+^ (neutrophils).

### Myelin visualization by luxol fast blue

To assess myelin clearance in the distal sciatic nerve segment, axotomized and sham-operated sciatic nerves from WT and *Ocm* KO mice were removed 7 d after transection and fixed by immersion in 4% PFA. The tissues were cryoprotected in 30% sucrose and embedded in Tissue-Tek O.C.T. compound. Staining with Luxol fast blue (Electron Microscopy Sciences) was performed on 20-μm cryostat sections. Briefly, nerves were incubated in H_2_O, 35% and 70% ethanol for 5 min each, followed by incubation in 0.1% Luxol fast blue overnight at 60°C. Nerves were subsequently destained in 0.05% lithium carbonate and incubated for 5 min each in increasing concentrations of ethanol (70%/95%/100%), followed by xylenes before images were captured using a light microscope. Positive myelin staining is expressed as a percentage of the total area examined. Images were captured at 20× magnification using Volocity software (PerkinElmer) and then quantified using MetaMorph. Three images per section were analyzed and averaged for each sample. Five samples per genotype were analyzed.

### Real-time PCR

The expression of three gp130 cytokines (*Lif*, *Cntf*, and *Il-6*) was analyzed by quantitative real-time PCR. Six hours and 9 d after unilateral sciatic nerve transection axotomized and sham-operated L3 and L4 DRG from WT and *Ocm* KO mice were removed, desheathed, and placed in RNAlater (Thermo Fisher Scientific). Two ganglia were pooled per sample. Three animals were included for each group. The tissue was homogenized, RNA was isolated, total RNA was measured, and 331 ng were reverse transcribed using a High Capacity cDNA Reverse Transcription kit (Thermo Fisher Scientific). Real-time PCR was performed in an ABI Step-One, using prevalidated TaqMan expression assays (Thermo Fisher Scientific) for *Lif* (Mm00434762), *Cntf* (Mm00446373), and *Il-6* (Mm00446190). Samples were assayed in triplicate and relative expression was determined using the comparative Ct method. mRNA values were normalized to the mRNA values for the internal control glyceraldehyde-3-phosphate dehydrogenase (GAPDH; Mm99999915) of the respective sham group for each genotype.

### Statistics

Experimenter’s performing data analysis were blinded to genotype. Data are expressed as mean ± SEM. Statistically significant differences were determined by a two-way ANOVA with a Tukey’s *post hoc* test using GraphPad Prism 9.2.0. Groups are considered statistically different if *p *<* *0.05. The number of samples per group is indicated in each figure legend.

## Results

### Effects of Ocm on the CL response examined in explant culture

**Figure 1.**
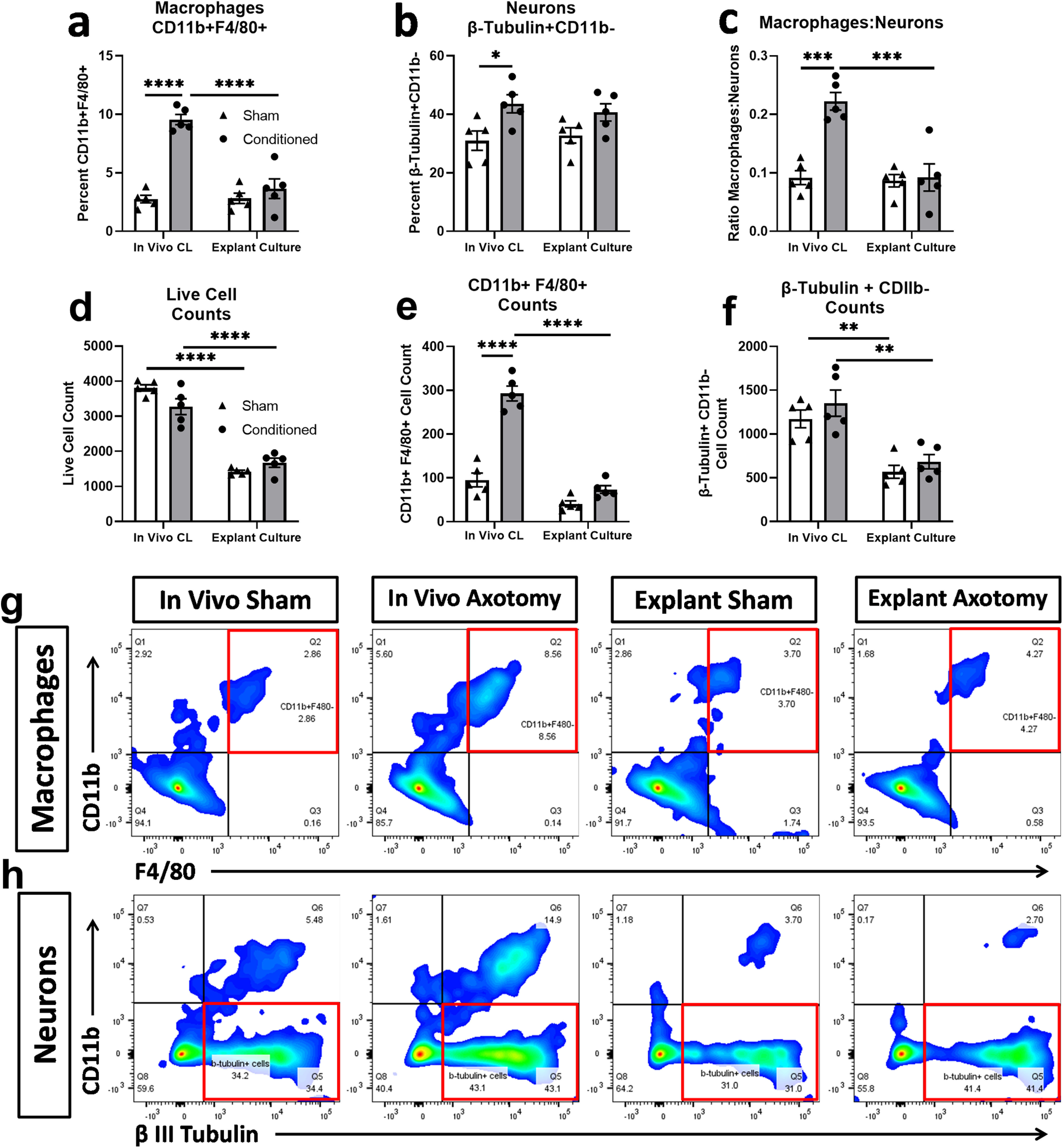
Relative macrophage content of DRGs *in vivo* and in explant culture after a CL. For *in vivo* studies, 7 d after a unilateral sciatic nerve transection or contralateral sham surgery, the nerves were crushed bilaterally and nerve regeneration was assayed 2 d later. For explant studies, DRGs were placed in explant culture for 2 d after unilateral sciatic nerve transection or contralateral sham surgery. The macrophage (***a***) and neuronal (***b***) content of each sample was determined by flow cytometry using two macrophage markers (CD11b and F4/80), one neuronal marker (β III Tubulin), and a live/dead cell stain. The ratio of macrophages to neurons is also given (***c***). Cell counts are also displayed for live cells (***d***) CD11b+ F4/80+ macrophages (***e***), and β-Tubulin+ CD11b– neurons (***f***). Representative heat maps are shown for CD11b and F4/80 (***g***) and CD11b and β III tubulin (***h***). Numbers in plots correspond to the percentage of total events in each quadrant. Events in quadrants outlined with a red box correspond to cells that are CD11b^+^F4/80^+^ (***g***, macrophages) or CD11b^–^ β III tubulin^+^ (***h***, neurons). *N* = 5/group. *p < 0.05, **p < 0.01, ***p < 0.001, ****p < 0.0001.

The increased growth capacity of sensory neurons as the result of a CL can be demonstrated in explant culture and *in vivo* (McQuarrie and Grafstein, 1973; McQuarrie et al., 1977; Edström et al., 1996; Shoemaker et al., 2005). To determine whether Ocm is involved in the CL response of DRG neurons, we began by examining the CL response measured in explant culture from WT and *Ocm* KO mice. Explant cultures enable the *in vitro* study of neurons while maintaining much of their *in vivo* tissue environment, including the presence of macrophages as shown in Figure 1*a*,*e*. The number of neurons was also determined for each sample (Fig. 1*b*,*f*), and the number of macrophages was also expressed per neuron (Fig. 1*c*).

**Figure 2.**
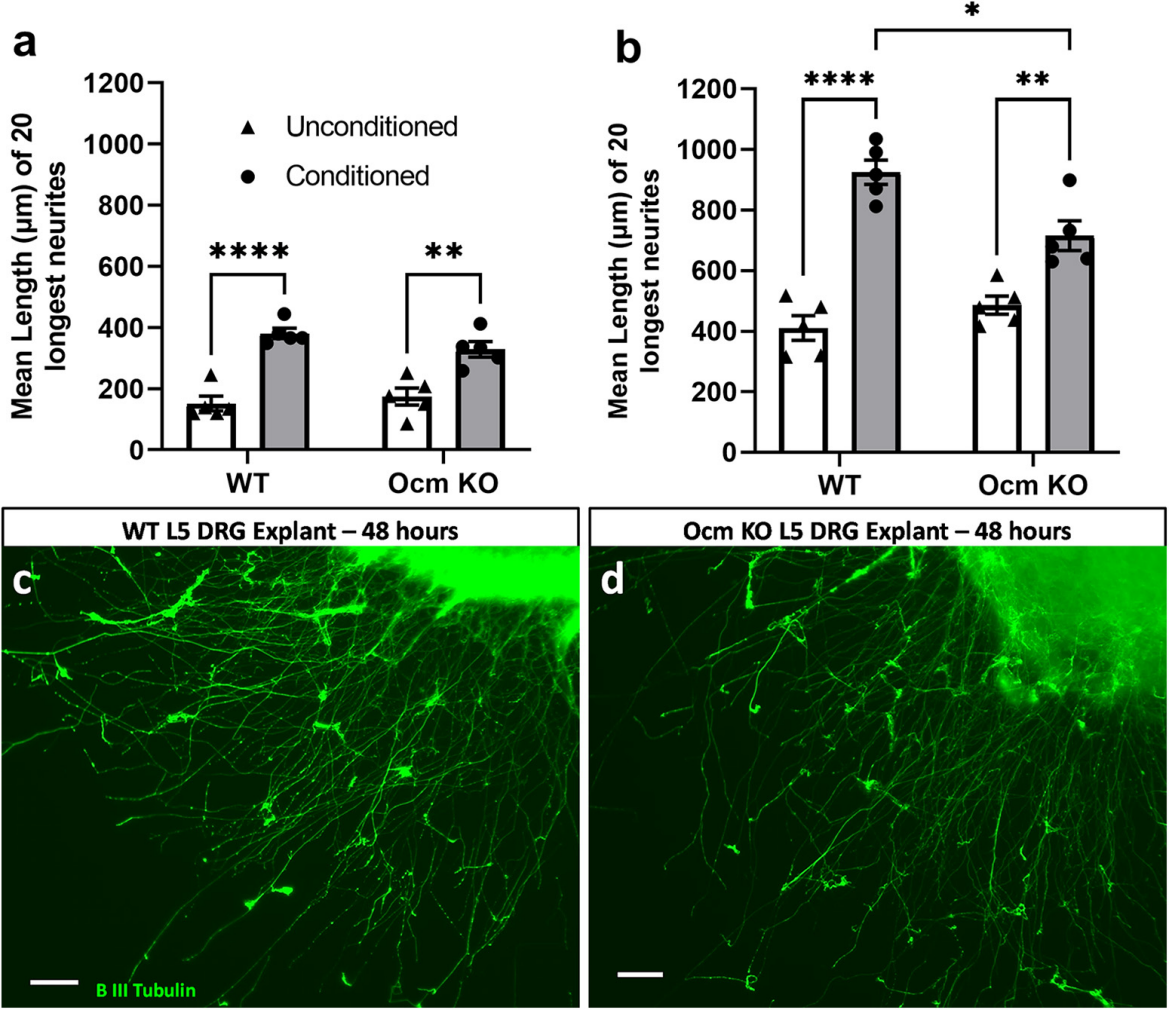
L5 DRG explants from both WT and *Ocm* KO mice exhibited a CL response at 24 h (***a***) and 48 h (***b***), although ganglia from *Ocm* KO mice showed a significantly smaller response than those from WT ganglia after 48 h in culture (***b***). Micrographs represent WT (***c***) and *Ocm* KO (***d***) ganglia following a CL and 48 h in culture. *N* = 5/group. Scale bars: 250 μm. *p < 0.05, **p < 0.01, ****p < 0.0001.

After both 24 and 48 h in culture, DRG explants from both WT and *Ocm* KO mice exhibited a CL response (Fig. 2*a*,*b*); however, the outgrowth from *Ocm* KO DRG in response to a CL was significantly smaller than that from WT DRG after 48 h in culture (Fig. 2*b–d*).

### Effects of Ocm on the CL response *in vivo*

**Figure 3.**
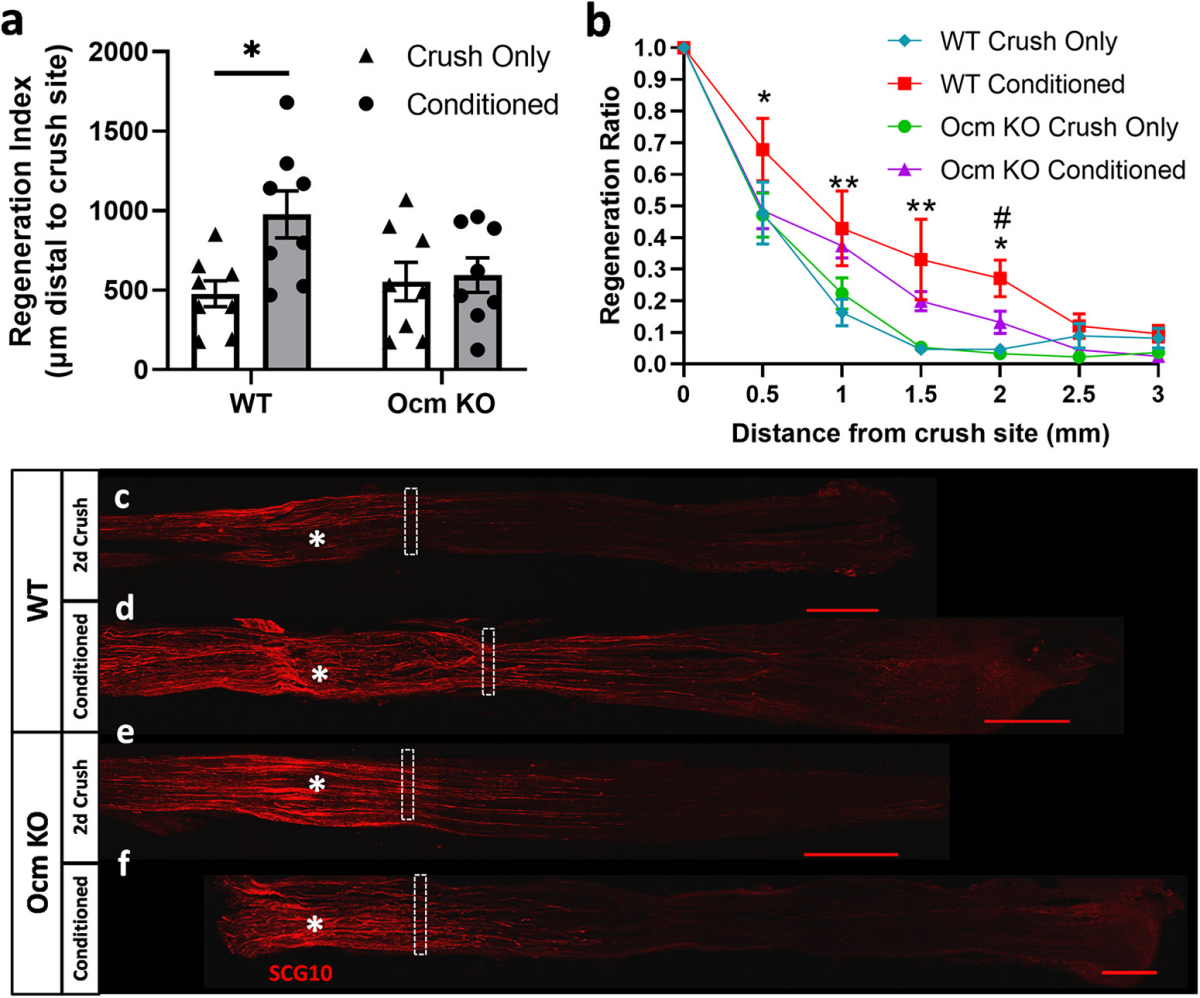
DRG from *Ocm* KO mice do not exhibit a CL response *in vivo*. Nine days after a CL and 2 d after a crush injury, axons from WT mice exhibit enhanced growth response *in vivo*, whereas axons from *Ocm* KO mice did not (***a***). The nerves were immunostained for SCG10. The regeneration index is the distance from the crush site to the site where the staining for SCG10 is half that seen at the crush site (***a*, *c–f***). The regeneration ratio is the ratio of staining at a site to that at the crush site. This ratio was determined for distances from 0.5 to 3.0 mm distal to the crush site (***b***). Images represent WT crush only (***c***) and conditioned plus crush (***d***) and *Ocm* KO crush only (***e***) and conditioned plus crush (***f***). Asterisks in the images indicate the crush site for each nerve. Dashed rectangles indicate where the immunostaining for SCG10 is reduced by 50% compared with the staining at the crush site. *N* = 8/group. Scale bars: 500 μm. In the line graph, *p < 0.05, **p < 0.01 comparing WT conditioned versus WT crush only. #*p* < 0.01 comparing WT conditioned versus *Ocm* KO conditioned.

We next looked at the CL response *in vivo* in WT and KO mice. Nine days after a CL (unilateral sciatic nerve transection) and 2 d after a test lesion (bilateral sciatic nerve crush at a site proximal to the site of transection), sciatic nerves were harvested, sectioned, and labeled with an antibody to SCG10. SCG10 is preferentially expressed in sensory axons, is rapidly downregulated distal to an injury site, and is highly expressed in regenerating fibers (Shin et al., 2012, 2014). The regeneration index identifies the distance from the crush site to the location where levels of SCG10 are half of their levels at the crush site, thus identifying the length to which approximately half of the axons have regenerated (Shin et al., 2014). Under these conditions, we found that sciatic nerves from WT mice exhibited a CL response while those from *Ocm* KO mice did not (Fig. 3*a*,*c–f*).

To examine further the distance the regenerating axons grew *in vivo* after a CL, a regenerative ratio was also determined by measuring the percent area stained from the site of the crush injury in 0.5-mm increments distally out to 3 mm (Fig. 3*b*). The data were displayed as a ratio of the percent area stained at each individual distance divided by the percent area stained at the crush site. WT conditioned nerves showed significantly more SCG10 staining at 0.5, 1, 1.5, and 2 mm distal to the crush site compared with WT crush only nerves, indicating that a CL increases the length of the axons regenerating *in vivo* (Fig. 3*b*). Nerves from *Ocm* KO mice did not show a significant difference in SCG10 staining in response to a CL at any distance distal to the crush site (Fig. 3*b*). Additionally, WT conditioned nerves showed significantly more SCG10 staining than *Ocm* KO conditioned nerves at 2 mm distal to the crush site (Fig. 3*b*,*d*,*f*). These data indicate that Ocm plays a prominent role *in vivo* in the CL response of DRG neurons. This *in vivo* experiment was repeated a second time, and similar results were obtained.

### Macrophage accumulation in the absence of Ocm

**Figure 4.**
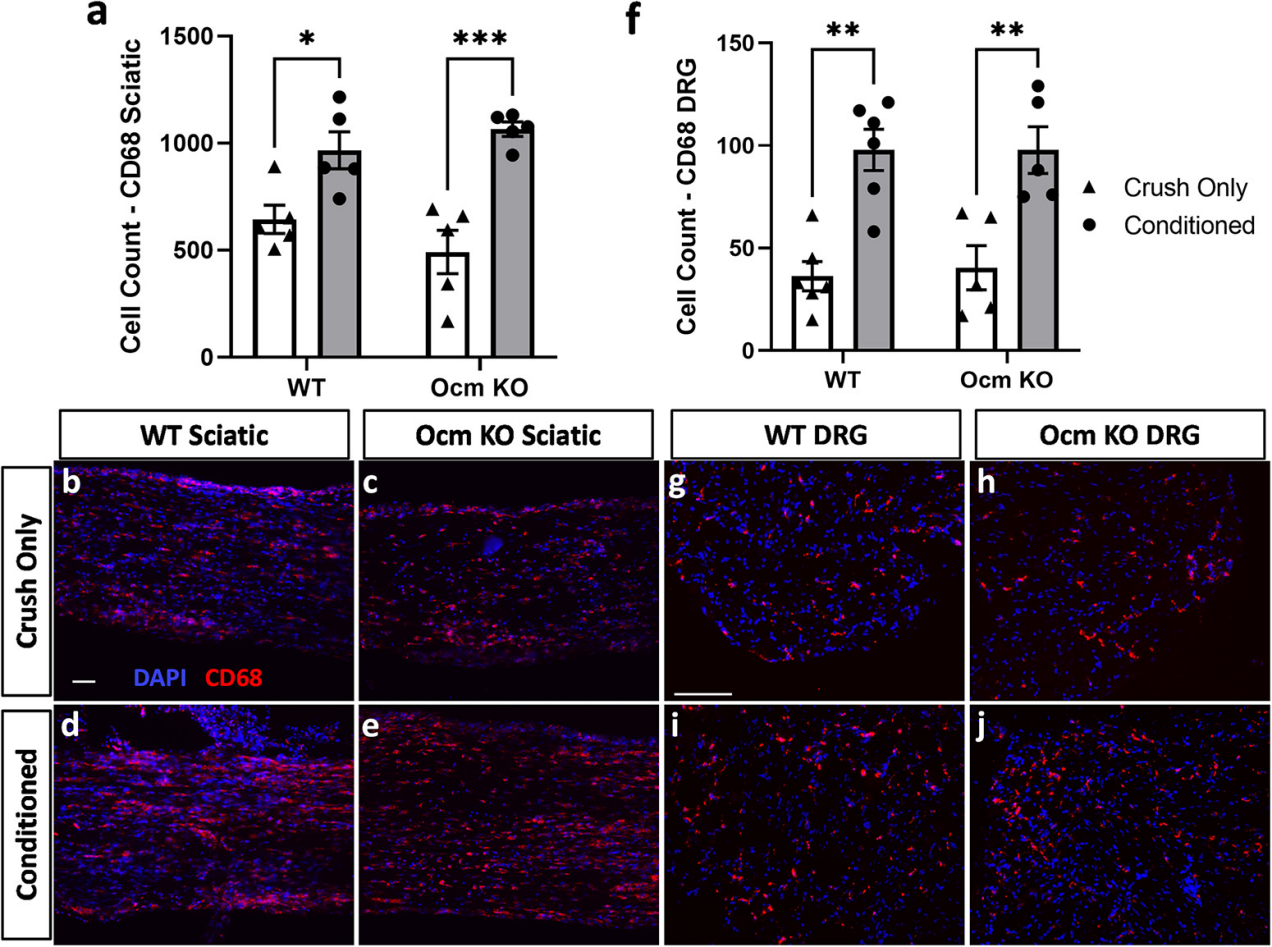
Macrophage accumulation in the distal sciatic nerve and DRG 2 d after a nerve crush with and without a prior CL in WT and *Ocm* KO mice. Macrophage accumulation observed by CD68 immunostaining in the distal sciatic nerve was similar after a CL in both genotypes (***a***). Macrophage accumulation was also increased in WT and Ocm KO DRG after a CL (***f***). Three adjacent fields from each sample were counted and summed. Images of WT crush only (***b***, ***g***) and conditioned plus crush (***d***, ***i***) and *Ocm* KO crush only (***c***, ***h***) and conditioned plus crush (***e***, ***j***). *N* = 5–6/group. Scale bars: 100 μm. *p < 0.05, **p < 0.01, ***p < 0.001.

In an attempt to explore how Ocm might facilitate nerve regeneration *in vivo*, we examined three factors known to influence regeneration: macrophage accumulation, Wallerian degeneration, and the induction of gp130 cytokines. The accumulation of macrophages in the distal nerve segment of the sciatic nerve after injury has been correlated with nerve regeneration (Bisby and Chen, 1990; Chen and Bisby, 1993; Dailey et al., 1998; Barrette et al., 2008). Therefore, we sought to determine whether Ocm might play a role in the accumulation of macrophages in the sciatic nerve after injury. Using immunohistochemistry, the number of CD68+ macrophages in the nerve was determined either 9 d after a transection (Conditioned) and 2 d after a crush injury, or just 2 d after a crush injury (Crush Only). We found that the CL produced a similar increase in CD68+ cells in the sciatic nerve from both WT and *Ocm* KO mice (Fig. 4*a–e*). These data indicate that *Ocm* does not influence macrophage accumulation in the distal nerve.

We also assessed the number of CD68+ macrophages in the DRG under the same conditions since macrophage accumulation in the ganglion is correlated with the CL response (Kwon et al., 2013; Niemi et al., 2013). There was a significant increase in CD68+ cells in conditioned DRG from both WT and *Ocm* KO mice compared to crush only (Fig. 4*f–j*).

**Figure 5.**
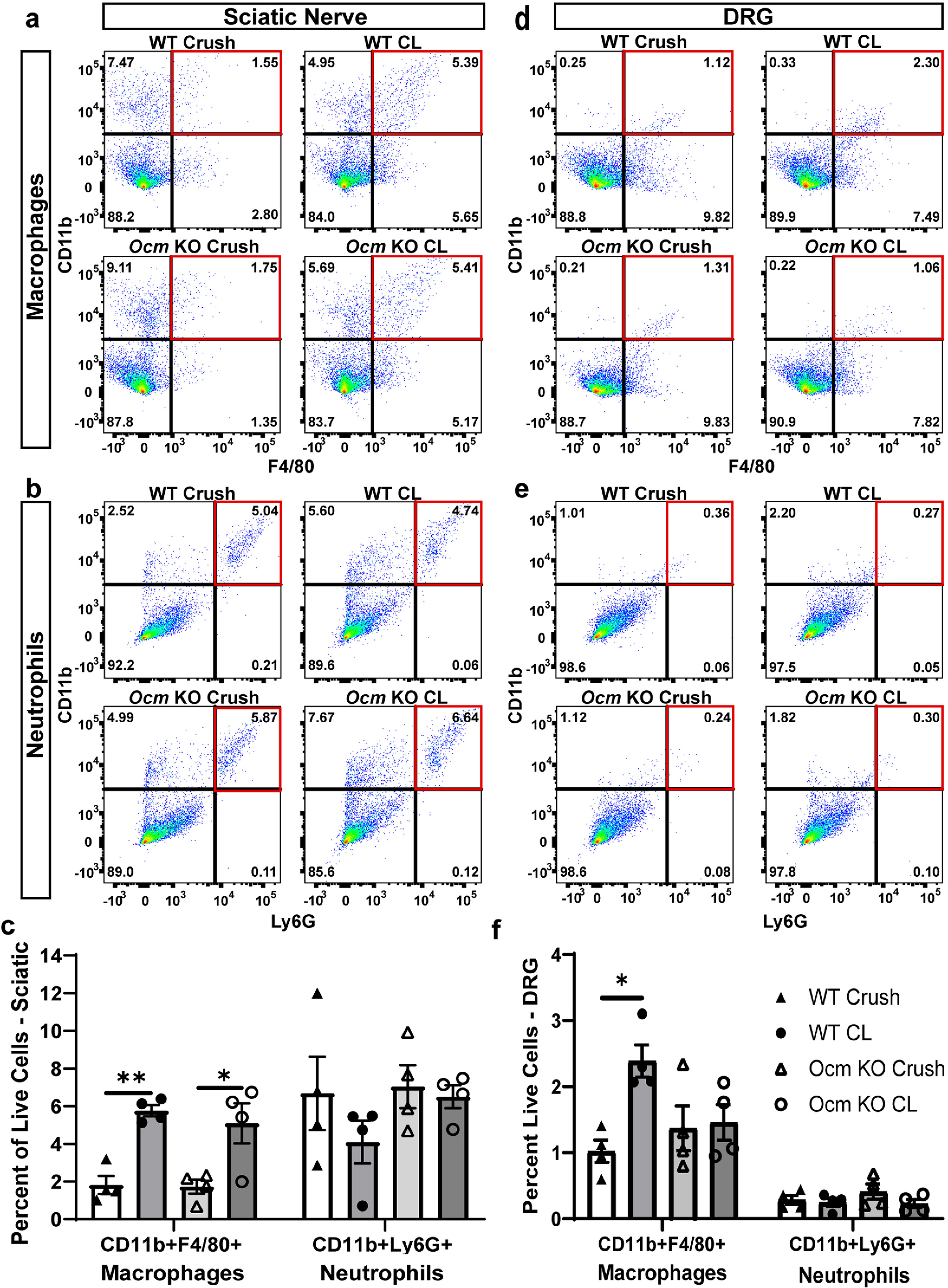
Flow cytometry studies on macrophage and neutrophil accumulation in the distal sciatic nerve and DRG in WT and *Ocm* KO mice after a nerve crush with and without a prior CL. Macrophages were determined by double staining with antibodies against CD11b and F4/80. Neutrophils were determined by double staining with antibodies against CD11b and Ly6G. For animals receiving a CL, the sciatic nerve was transected unilaterally 7 d before the nerve was crushed (samples labeled CL). Forty-eight hours later, the ipsilateral distal sciatic nerve and the ipsilateral DRG were taken for flow cytometry. The contralateral sciatic nerve received a crush only (samples labeled Crush). Representative dot plots for macrophages (***a***, ***d***) and neutrophils (***b***, ***e***) in the sciatic nerve (***a***, ***b***) and DRG (***d***, ***e***). Numbers in plots correspond to the percentage of total events in each quadrant. Events in quadrant outlined with a red box correspond to cells that are CD11b^+^F4/80^+^ (***a***, ***d***, macrophages) or CD11b^+^Ly6G^+^ (***b***, ***e***, neutrophils). Bar graphs indicate mean percent CD11b^+^F4/80^+^ and CD11b^+^Ly6G^+^ events in the sciatic nerve (***c***) and DRG (***f***). *n* = 4 animals per condition per genotype. *p < 0.05, **p < 0.01.

To obtain a more quantitative measure of macrophage accumulation, CD11b+F4/80+ macrophages were examined in the sciatic nerve and DRG using flow cytometry. The results obtained were similar to those obtained with immunohistochemistry. No difference in macrophage presence in the sciatic nerve was found between genotypes after a CL (Fig. 5*a*,*c*), but an increase in macrophage accumulation in the DRG was found after a CL in WT but not in *Ocm* KO mice (Fig. 5*d*,*f*).

Neutrophils were identified by being positive for CD11b and Ly6G. Such cells are undetectable in the sciatic nerve from sham-operated mice (Lindborg et al., 2017). Following axotomy, no significant differences were found in the sciatic nerve between genotypes or between crush only and a CL followed by crush (Fig. 5*b*,*c*). As reported previously (Lindborg et al., 2018), no significant number of neutrophils were found in the DRG from sham-operated or lesioned animals (Fig. 5*e*,*f*).

### Clearance of myelin in the absence of Ocm

**Figure 6.**
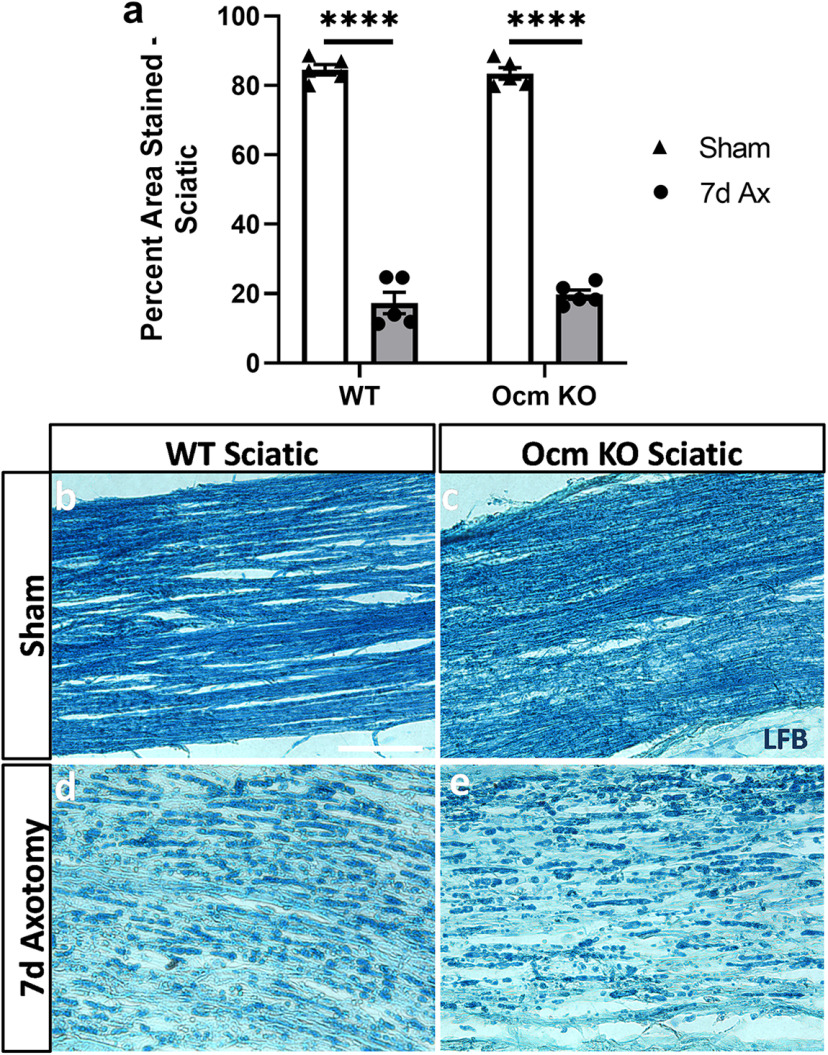
Myelin clearance from the distal sciatic nerve from WT and *Ocm* KO mice. Seven days after unilateral sciatic nerve transection changes in myelin clearance were determined by staining with luxol fast blue. Transected distal nerve segments from WT and *Ocm* KO mice showed significantly less staining than the sham-operated contralateral nerves, and no difference was seen between genotypes (***a***). The micrographs represent sections of WT sham-operated nerves (***b***) and transected distal nerves (***d***) and sections of *Ocm* KO sham-operated nerves (***c***) and transected distal segments (***e***). *N* = 5/group. Scale bar: 100 μm. ****p < 0.0001.

Myelin clearance from the distal nerve after axotomy is important in the peripheral nervous system for subsequent regeneration (Barrette et al., 2008). Therefore, we examined myelin clearance in WT and *Ocm* KO mice. The clearance of myelin in the distal sciatic nerve was assessed by staining with luxol fast blue. At 7 d after sciatic nerve transection, luxol fast blue staining was reduced to the same extent in nerves from WT and *Ocm* KO mice (Fig. 6). These results indicate that Ocm is not exerting its proregenerative effects by influencing myelin clearance.

### Effects of Ocm on expression of gp130 cytokines

**Figure 7.**
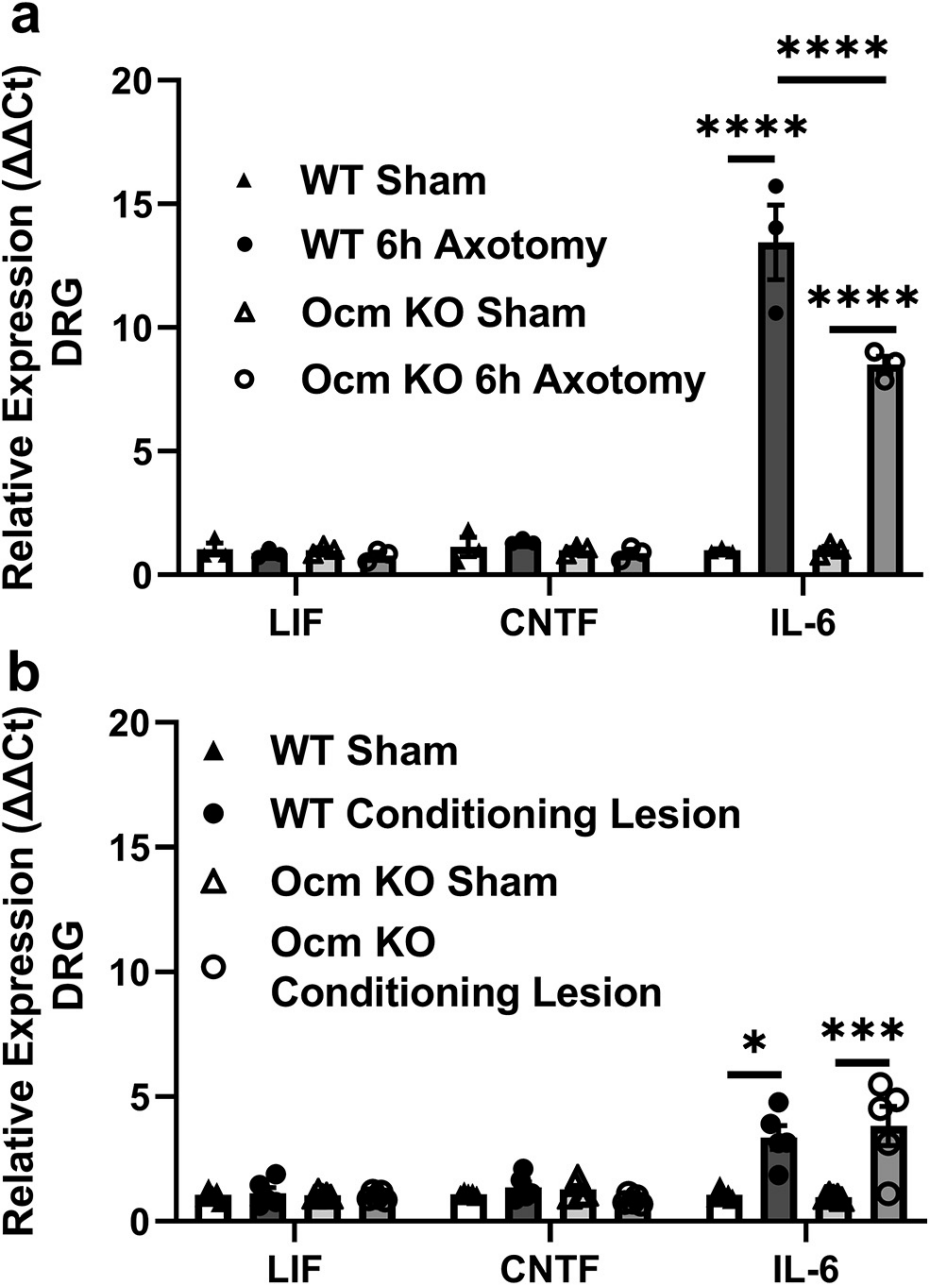
*Il-6* mRNA expression is upregulated in the DRG at various timepoints after sciatic nerve injury. Although an increase was seen in DRG from both WT and *Ocm* KO mice 6 h after sciatic nerve transection, the increase was significantly larger in the former (***a***). *Il-6* mRNA expression was not different between WT and Ocm KO mice following a CL compared with sham-operated contralateral DRG (***b***). *Lif* and *Cntf* mRNA was not found to increase with injury in pooled lumbar DRG 6 h after a sciatic nerve transection (***a***) or a CL (***b***). *N* = 3–5/group. *p < 0.05, ***p < 0.001, ****p < 0.0001.

It has been shown that axotomy influences expression of gp130 cytokines in peripheral ganglia (for review, see Zigmond, 2012). Although LIF is expressed in the superior cervical ganglion after axotomy, it is not expressed in the axotomized DRG at times examined thus far (Sun and Zigmond, 1996; Thompson et al., 1997). CNTF is expressed in myelinating Schwann cells in the intact sciatic nerve (Dobrea et al., 1992; Rende et al., 1992), but expression actually decreases after nerve transection (Friedman et al., 1992; Sendtner et al., 1992; Seniuk et al., 1992). CNTF was not found in neurons in the DRG *in vivo* though it is expressed in cultured neurons (Sango et al., 2007). *Il-6* mRNA expression is increased in DRG after axotomy (Murphy et al., 1995). There is a disagreement in the literature as to whether there is a CL response in DRG neurons from *Il-6 K*O mice (Cafferty et al., 2004; Cao et al., 2006). We looked at mRNA expression of these three gp130 cytokines in the DRG 6 h after a sciatic nerve transection (Fig. 7*a*) and 7 d after a CL followed by 2 d after a nerve crush (Fig. 7*b*). Only *Il-6* expression was affected differentially in WT and *Ocm* KO animals at the 6-h time point. Although *Il-6* increased after axotomy in ganglia from both genotypes, the increase was significantly less in DRG from *Ocm* KO mice compared with that from WT mice (Fig. 7).

## Discussion

Our study is the first to look at nerve regeneration in *Ocm* KO mice. Ocm was proposed as a promoter of nerve regeneration based on studies on regeneration of retinal ganglion cell axons following induced ocular inflammation in male Fischer rats (Yin et al., 2006). Benowitz and colleagues reported Ocm protein in the eye and showed that the stimulation of regeneration caused by inflammation could be blocked by reagents that antagonize the action of Ocm (Yin et al., 2009). Fischer and colleagues have presented data supporting a different view of how inflammation leads to the growth of retinal ganglion axons. Working with female Sprague Dawley rats and female C57BL/6 mice, they reported that the stimulation of regeneration by inflammation is mediated by the release of gp130 cytokines (i.e., CNTF, LIF, and IL-6) from retinal astrocytes (Hauk et al., 2008; Leibinger et al., 2009, 2013).

We find that the CL effect measured in sensory neurons *in vivo* is diminished in *Ocm* KO mice. In addition, the CL effect measured in explanted DRG was inhibited in KO mice, though it was not completely blocked. Obviously, the *in vivo* experiment is the most physiologically relevant and makes no assumptions as to the cellular site of action of Ocm. Thus, Ocm might act in the ganglion or in the proximal or distal transected nerve.

It should be noted that in our experiments, we have performed a unilateral CL and then compared the effects in the ipsilateral to those in the contralateral DRGs. This comparison may underestimate the magnitude of the effect on the ipsilateral ganglia, as in recent studies in the rat, it has been shown that, after spinal nerve transection, some stimulation of gene expression and neurite outgrowth occurs not only in the ipsilateral DRGs but also, though to a lesser extent, in the contralateral DRGs (Hasmatali et al., 2019, 2020; Verge et al., 2020).

Ocm binds to retinal ganglion cells *in vivo*; however, whether this is also true for cells in the DRG, either neuronal or non-neuronal, is not known. Additionally, an Ocm receptor and an exact signaling pathway in the nervous system have not been defined. The growth effect of Ocm on cultured retinal ganglion cells is completely blocked by the transcriptional inhibitor actinomycin D (Yin et al., 2006). In an earlier study, the CL effect on DRG neurons was similarly shown to be blocked by a different transcriptional inhibitor, 5,6-dichlorobenzimidazole riboside (Smith and Skene, 1997). Our results raise the possibility that Ocm promotes regeneration of sensory neurons in part by altering neuronal gene expression, specifically the expression of the gp130 cytokine IL-6. *IL-6* mRNA has been shown to increase in DRG neurons after axotomy (Murphy et al., 1995). Cafferty et al. (2004) showed that addition of IL-6 to DRG cultures increased neurite outgrowth, and Dubový et al. (2019) showed that intrathecal injection of IL-6 produced a CL-like response after peripheral nerve injury. In the present study, we found that *Il-6* mRNA was also increased in DRG from *Ocm* KO mice at 6 h, but to a significantly lesser extent than that from WT animals (Fig. 7). LIF and CNTF, two other members of this cytokine family, were neither induced in the DRG by axotomy nor altered by the absence of Ocm (Fig. 7). Although the time point analyzed here (6 h) is before hematogenous macrophage accumulation in the DRG occurs, it is possible that resident macrophages also express Ocm. In addition, neutrophils, which can express Ocm, have been shown to accumulate already in the sciatic nerve when examined 8 h after partial nerve injury (Perkins and Tracey, 2000). The possibility that Ocm alters the expression of IL-6 offers a partial resolution of the conflicting views proposed by the Benowitz and Fischer groups described above on whether Ocm or gp130 cytokines mediate the effects of inflammation on optic nerve regeneration.

A finding that was quite unexpected in our study was that accumulation of macrophages in the DRG, though not in the sciatic nerve, was inhibited in the *Ocm* KO animals (Figs. 4, 5). This finding raises the possibility that Ocm has chemotactic activity or that it can modulate the expression of monocyte chemokines (e.g., CCL2) or their signaling within the DRG. Under conditions in which macrophage accumulation in the DRG is inhibited, the CL effect is blocked (Niemi et al., 2013).

Often a neuron’s ability to initiate axonal growth after an injury is distinguished from its ability to elongate that axon to reach its target tissues. Here, we observed significantly less outgrowth *in vitro* from explants of *Ocm* KO compared with WT mice at 48 h, but not at 24 h, indicating that Ocm may be involved in elongation of regenerating axons rather than initiation of outgrowth.

To promote regeneration and functional recovery, it is likely that a combinatorial approach will be most effective (Benowitz and Popovich, 2011). Prior studies of Ocm have indicated that Ocm can exert its growth-promoting effects in coordination with other agents. For example, it has been shown that increasing retinal levels of Ocm and decreasing PTEN expression together can be used to promote regeneration after optic nerve crush (de Lima et al., 2012). In addition, the fact that elevated cAMP levels are required for the proregenerative effects of Ocm indicates that there may be other players involved.

In summary, using the *Ocm* KO mouse, we have demonstrated that this cytokine is necessary for the normal CL response to occur and that this effect could be because of a decrease in the axotomy-induced expression of IL-6 or another macrophage cytokine.

## Synthesis

Reviewing Editor: Jeffery Twiss, University of South Carolina Decisions are customarily a result of the Reviewing Editor and the peer reviewers coming together and discussing their recommendations until a consensus is reached. When revisions are invited, a fact-based synthesis statement explaining their decision and outlining what is needed to prepare a revision will be listed below. The following reviewer(s) agreed to reveal their identity: NONE.

The reviewers and reviewing editor felt that your manuscript addresses an important area of investigation as it is still unclear what the major mechanisms are that underlie the conditioning lesion response. Ocm has been shown to contribute to CNS axon regeneration in an optic nerve crush model where inflammation is involved, but the role in PNS regeneration has not been completely clear. The use of the conditional Ocm knockout animal provides a convincing test for Ocm contribution to the conditioning lesion. However, reveiwers also noted that while the combination of in vivo and in vitro approaches strengthens the findings that Ocm is involved in the conditioning lesion response, there are areas of the manuscript that need additional insight or discussion as detailed below.

Major Issues:

1. The use of contralateral DRG as a control is not appropriate. It has been shown that DRG contralateral to injury are in a heightened state of plasticity at 7 days post sciatic nerve injury and thus, the findings in the current paper are actually likely underestimated (Hasmatali et al., JNEN 2019; Hasmatali et al., 2020 NRR) and there are systemic responses with respect to elevation of circulating cyctokines that may factor into this (Verge et al., NRR 2020). A unilateral crush injury on new control mice should have been included and naïve not contralateral DRG should have been used in addition to the sham. At the least, the authors should provide a discussion of these points as the magnitude of Ocm’s effects reported here may be underestimated by use of contralateral control.

2. The assessment of cytokines and inflammatory markers (including oncomodulin) should have also been done at one week post-injury and not just the early 6 hr timepoint, as this would reveal the state of the environment at the end of the conditioning period prior to the crush injury. This is important as it has been shown in Sprague Dawley rats that the expression of the markers examined in this paper and more importantly oncomodulin within the first week of injury peaks at the 7 day point (Kwon et al., J Neurosci 2013). Measuring this would also confirm whether this is still the case for Ocm in the wild type mice, an absence of Ocm confirm the KO and also confirm the peak expression of the cytokines examined.

3. The conclusion that Ocm signaling through Il6 promotes axon regeneration would be strengthened by a rescue experiment in the Ocm KO neurons. Specifically, overexpresssing IL6 or using exogenous IL6 would address this using WT/KO explant cultures and assay axon growth as shown in Figure 2. In the online disucssion it was pointed out that this would obviously require that the IL-6 receptor is there as well in the culture condition. This can quickly be tested by RT-PCR with appropriate choice of controls. Given that there is an initial peak in IL-6 expression and then another at 7 days adding it to conditioned explants in culture would likely suffice.

4. There was a very interesting observation of a significant increase in % of cells that are neurons in the CL in vivo group. Does this reflect increased neuronal DRG numbers from the in vivo experiments in Figure 1 or a shift in the proportional representation? The results should reported in the body of the results along with the findings for the macrophages. Reporting the absolute number of neurons rather than percentages could resolve this issue.

5. Please give more information on the creation of the KO mouse used in these experiments, so that the reader does not have to go to the Tong et al., reference.

6. In Figures 1 & 2, it is not made clear in the results section or figure legends whether the unconditioned underwent a 2 day crush injury prior to assay or in vivo - please clarify. Representative imageswould strengthen Figure 1.

7. In Figure 1, the authors show that there is no axotomy mediated increase in macrophage numbers in explant cultures. If so, it is not clear why there is no difference in axon growth between WT vs. KO in unconditioned neurons in Figure 2?

8. Although no difference was shown quantitatively between WT and KO mice with respect to myelin clearance, the representative pictures show far lees myelin debris as a % of the area covered in. the WT section.

9. In figure 4, the authors mention that a large s.e.m. may account for the lack of a significant change in macrophage #s in the DRG? Why were only 3 animals included in this assessment and not the 5 as for the nerves? This may have been just underpowered. Morphologically, did the CD68+ cells look different between WT and Ko mice? The reviewers strongly recommended increasing the N for this experiment.

Minor issues:

1. Please check Figure 1 legend for spelling errors;

2. Figure 7 legend makes does not reference all markers examined.

## Author Response

Dear Dr. Twiss,

We are grateful for the comments of the reviewers and believe the revisions we have made have significantly strengthened our manuscript. Below, I have copied the reviewers’ comments and our responses are listed below each comment. In the manuscript, our major changes are shown in blue font.

Major Issues:

1. The use of contralateral DRG as a control is not appropriate. It has been shown that DRG contralateral to injury are in a heightened state of plasticity at 7 days post sciatic nerve injury and thus, the findings in the current paper are actually likely underestimated (Hasmatali et al., JNEN 2019; Hasmatali et al., 2020 NRR) and there are systemic responses with respect to elevation of circulating cytokines that may factor into this (Verge et al., NRR 2020). A unilateral crush injury on new control mice should have been included and naïve not contralateral DRG should have been used in addition to the sham. At the least, the authors should provide a discussion of these points as the magnitude of Ocm’s effects reported here may be underestimated by use of contralateral control.

Authors’ Response:

We thank the reviewers for these important comments. In our experiments, we measured regeneration either in explant culture or in vivo. As is now more clearly described in our revised manuscript, in both types of experiments, the sciatic nerve was either transected unilaterally or received a sham procedure in which the nerve was just exposed. For explants, 7 d later, the DRGs ipsilateral or contralateral were dissected (thus cutting their axons for the first or second time), placed in explant culture for 48 h, and then neurite outgrowth was measured. For the in vivo experiments, 7 d after the unilateral conditioning lesion, the sciatic nerve was crushed proximally on both sides of the animals, and 2 d later axonal regeneration was measured from the crush site. As correctly pointed out by the reviewers, since we compared growth in ipsilateral ganglia to that in contralateral ganglia, we ignored the possibility that there might be enhanced growth contralateral to a unilateral conditioning lesion, and, if that did occur, that could lead to an underestimation of the effect on the ipsilateral side. As suggested by the reviewers, we have added a paragraph discussing this possibility to our Discussion section.

Such contralateral phenomena were pointed out by Steve McMahon and colleagues in 1999 in a paper entitled “Does the right side know what the left is doing” (Koltzenburg et al., 1999). More recently, as indicated by the reviewers, new aspects of such phenomena have been reported by the Verge/Misra laboratories (Hasmatali et al., 2019; Hasmatali et al., 2020; Verge et al., 2020). Their studies differ from ours in two ways, and these may or may not be relevant. First, their experiments were done in male Wistar rats rather than in male and female CBA mice as ours were. Second, the nature of the lesion was quite different. Hasmatali et al. transected the sciatic nerve at the level of the L4-L6 spinal nerves so as to ensure “nearly 100% injury of the L4-L6 DRG neurons” (Hasmatali et al., 2019; Hasmatali et al., 2020). In contrast, we transected the sciatic nerve at mid-thigh level, which only leads to axotomy of approximately 50% of the neurons in these ganglia (Renthal et al., 2020). In addition, lesioning at the level of the spinal nerves axotomizes DRG neurons considerably closer to the cell bodies than does lesioning at the sciatic nerve, which could affect the magnitude of certain phenotypic changes (Lieberman, 1971).

The article by Verge et al. (2020) cites another recent relevant paper, namely that by Dubový et al. (2019). We have been quite perplexed by the latter’s study. Unlike the studies of Hasmatali et al., Dubovy et al. not only report changes in gene expression (i.e., GAP 43 and SCG10) in the contralateral ganglion, but also report that the changes are quantitatively the same in ipsilateral and contralateral ganglion. This implies that, whatever mechanism(s) is responsible for contralateral effects (perhaps trans-synaptic effects in the spinal cord or systemic effects), they are equally powerful to the effects of axotomy itself. This is not our experience (e.g., see Fig. 6; Hyatt-Sachs et al., 1993).

2. The assessment of cytokines and inflammatory markers (including oncomodulin) should have also been done at one week post-injury and not just the early 6 hr time point, as this would reveal the state of the environment at the end of the conditioning period prior to the crush injury. This is important as it has been shown in Sprague Dawley rats that the expression of the markers examined in this paper and more importantly oncomodulin within the first week of injury peaks at the 7 day point (Kwon et al., J Neurosci 2013). Measuring this would also confirm whether this is still the case for Ocm in the wild type mice, an absence of Ocm confirm the KO and also confirm the peak expression of the cytokines examined.

Authors’ Response:

As suggested by the reviewers, we now report qPCR data for IL-6 at both 6 h post-injury and 7 d after conditioning lesion followed by a 2 d crush injury.

3. The conclusion that Ocm signaling through Il6 promotes axon regeneration would be strengthened by a rescue experiment in the Ocm KO neurons. Specifically, overexpressing IL6 or using exogenous IL6 would address this using WT/KO explant cultures and assay axon growth as shown in Figure 2. In the online discussion it was pointed out that this would obviously require that the IL-6 receptor is there as well in the culture condition. This can quickly be tested by RT-PCR with appropriate choice of controls. Given that there is an initial peak in IL-6 expression and then another at 7 days adding it to conditioned explants in culture would likely suffice.

Authors’ Response:

From the literature there is ample evidence that IL-6 can produce a condition lesioning-like effect in culture that does not require addition of the IL-6 soluble receptor (e.g., Cafferty et al., 2004). On the other hand, there is a controversy as to results with IL-6 knockout mice with Cafferty et al. (2004) reporting that IL-6 plays a necessary role in the conditioning lesion response, and Cao et al. (2006) finding that it does not. More recently, Dubový et al. (2019) showed that intrathecal administration of IL-6 produced a conditioning lesion-like effect, in their case in the ulnar nerve. Thus, whereas IL-6 is known to increase neurite outgrowth in culture and regeneration in vivo, this does not prove oncomodulin produces its effect by stimulating IL-6 expression. We have toned down our claims as to the implications of our IL-6 data.

4. There was a very interesting observation of a significant increase in % of cells that are neurons in the CL in vivo group. Does this reflect increased neuronal DRG numbers from the in vivo experiments in Figure 1 or a shift in the proportional representation? The results should reported in the body of the results along with the findings for the macrophages. Reporting the absolute number of neurons rather than percentages could resolve this issue.

Authors’ Response:

As requested, we now report the data in Fig. 1 both as a change in the percentages and in the absolute numbers of macrophages and neurons.

5. Please give more information on the creation of the KO mouse used in these experiments, so that the reader does not have to go to the Tong et al., reference.

Authors’ Response:

In the Materials and Methods section, we now have included more information on the creation of the oncomodulin KO mouse as requested.

6. In Figures 1 & 2, it is not made clear in the results section or figure legends whether the unconditioned underwent a 2 day crush injury prior to assay or in vivo - please clarify. Representative images would strengthen Figure 1.

Authors’ Response:

In the in vivo experiments, as we now emphasize both in the Results section and the figure legends, both conditioned and unconditioned mice received a 2 d crush prior to examining regeneration. For the explant studies, none of the mice received a 2 d crush. Instead all the DRGs were axotomized (either for the first time or the second time) as a consequence of the dissection and explantation of the ganglia and neurite outgrowth was measured 2 d later.

The reason there are no images shown with Fig. 1 is that these are flow cytometry data not immunohistochemical data. Micrographs are shown in Figs. 2 & 4.

7. In Figure 1, the authors show that there is no axotomy mediated increase in macrophage numbers in explant cultures. If so, it is not clear why there is no difference in axon growth between WT vs. KO in unconditioned neurons in Figure 2?

Authors’ Response:

As we now discuss, we think that the fact that there is no axotomy-induced increase in macrophages in explant cultures reflects either (or perhaps both) the turnover of macrophages in culture with no further infiltration from the circulation or that macrophages migrate outside of the explant, as we have previously observed for glial cells.

We do not understand the basis of the question as to “why there is no difference in axon growth between WT vs. KO in unconditioned neurons in Figure 2?"

8. Although no difference was shown quantitatively between WT and KO mice with respect to myelin clearance, the representative pictures show far less myelin debris as a % of the area covered in the WT section.

Authors’ Response:

Having searched through our images, we have now substituted images in Fig. 7 of LFB staining that are more representative of the mean values.

9. In figure 4, the authors mention that a large s.e.m. may account for the lack of a significant change in macrophage #s in the DRG? Why were only 3 animals included in this assessment and not the 5 as for the nerves? This may have been just underpowered. Morphologically, did the CD68+ cells look different between WT and KO mice? The reviewers strongly recommended increasing the N for this experiment.

Authors’ Response:

Thank you for pointing out that only 3 ganglia per animals were included in Fig. 4. This was a lapse on our part. We now show the data for 5-6 DRGs per group.

Minor issues:

1. Please check Figure 1 legend for spelling errors;

Authors’ Response:

We apologize for the sloppiness. The errors have been corrected.

2. Figure 7 legend makes does not reference all markers examined.

Authors’ Response:

This is now corrected.

References

Cafferty WB, Gardiner NJ, Das P, Qiu J, McMahon SB, Thompson SW (2004) Conditioning injury-induced spinal axon regeneration fails in interleukin-6 knock-out mice. J Neurosci 24:4432-4443.

Cao Z, Gao Y, Bryson JB, Hou J, Chaudhry N, Siddiq M, Martinez J, Spencer T, Carmel J, Hart RB, Filbin MT (2006) The cytokine interleukin-6 is sufficient but not necessary to mimic the peripheral conditioning lesion effect on axonal growth. J Neurosci 26:5565-5573.

Dubový P, Klusáková I, Hradilová-Svíženská I, Brázda V, Kohoutková M, Joukal M (2019) A conditioning sciatic nerve lesion triggers a pro-regenerative state in primary sensory neurons also of dorsal root ganglia non-associated with the damaged nerve. Front Cell Neurosci 13:11.

Hasmatali JCD, De Guzman J, Johnston JM, Noyan H, Juurlink BH, Misra V, Verge VMK (2020) FOXO3a as a sensor of unilateral nerve injury in sensory neurons ipsilateral, contralateral and remote to injury. Neural Regen Res 15:2353-2361.

Hasmatali JCD, De Guzman J, Zhai R, Yang L, McLean NA, Hutchinson C, Johnston JM, Misra V, Verge VMK (2019) Axotomy induces phasic alterations in luman/CREB3 expression and nuclear localization in injured and contralateral uninjured sensory neurons: correlation with intrinsic axon growth capacity. J Neuropathol Exp Neurol 78:348-364.

Hyatt-Sachs H, Schreiber RC, Bennett TA, Zigmond RE (1993) Phenotypic plasticity in adult sympathetic ganglia in vivo: effects of deafferentation and axotomy on the expression of vasoactive intestinal peptide. J Neurosci 13:1642-1653.

Koltzenburg M, Wall PD, McMahon SB (1999) Does the right side know what the left is doing? Trends Neurosci 22:122-127.

Lieberman AR (1971) The axon reaction: a review of the principle features of perikaryal responses to axon injury. In: International Review of Neurobiology (Pfeiffer CC, Smythies JR, eds), pp 49-124. New York: Academic Press.

Renthal W, Tochitsky I, Yang L, Cheng YC, Li E, Kawaguchi R, Geschwind DH, Woolf CJ (2020) Transcriptional Reprogramming of Distinct Peripheral Sensory Neuron Subtypes after Axonal Injury. Neuron 108:128-144.e129.

Verge VMK, Hasmatali JCD, Misra V (2020) When the left side knows something happened to the right - sensing injury in neurons contralateral and remote to injury. Neural Regen Res 15:1854-1855.
